# Capsaicin Induces “Brite” Phenotype in Differentiating 3T3-L1 Preadipocytes

**DOI:** 10.1371/journal.pone.0103093

**Published:** 2014-07-29

**Authors:** Ritesh K. Baboota, Dhirendra P. Singh, Siddhartha M. Sarma, Jaspreet Kaur, Rajat Sandhir, Ravneet K. Boparai, Kanthi K. Kondepudi, Mahendra Bishnoi

**Affiliations:** 1 National Agri-Food Biotechnology Institute (NABI), SAS Nagar, Punjab, India; 2 Department of Biotechnology, University Institute of Engineering and Technology (UIET), Panjab University, Chandigarh, India; 3 Department of Biochemistry, Panjab University, Chandigarh, India; Complexo Hospitalario Universitario de Santiago, Spain

## Abstract

**Objective:**

Targeting the energy storing white adipose tissue (WAT) by pharmacological and dietary means in order to promote its conversion to energy expending “brite” cell type holds promise as an anti-obesity approach. Present study was designed to investigate/revisit the effect of capsaicin on adipogenic differentiation with special reference to induction of “brite” phenotype during differentiation of 3T3-L1 preadipocytes.

**Methods:**

Multiple techniques such as Ca^2+^ influx assay, Oil Red-O staining, nutrigenomic analysis in preadipocytes and matured adipocytes have been employed to understand the effect of capsaicin at different doses. In addition to *in-vitro* experiments, *in-vivo* studies were carried out in high-fat diet (HFD) fed rats treated with resiniferatoxin (RTX) (a *TRPV1* agonist) and in mice administered capsaicin.

**Results:**

*TRPV1* channels are expressed in preadipocytes but not in adipocytes. In preadipocytes, both capsaicin and RTX stimulate Ca^2+^ influx in dose-dependent manner. This stimulation may be prevented by capsazepine, a *TRPV1* antagonist. At lower doses, capsaicin inhibits lipid accumulation and stimulates *TRPV1* gene expression, while at higher doses it enhances accumulation of lipids and suppresses expression of its receptor. In doses of 0.1–100 µM, capsaicin promotes expression of major pro-adipogenic factor *PPARγ* and some of its downstream targets. In concentrations of 1 µM, capsaicin up-regulates anti-adipogenic genes. Low-dose capsaicin treatment of 3T3-L1 preadipocytes differentiating into adipocytes results in increased expression of brown fat cell marker genes. In white adipose of mice, capsaicin administration leads to increase in browning-specific genes. Global *TRPV1* ablation (i.p. by RTX administration) leads to increase in locomotor activity with no change in body weight.

**Conclusion:**

Our findings suggest the dual modulatory role of capsaicin in adipogenesis. Capsaicin inhibits adipogenesis in 3T3-L1 *via TRPV1* activation and induces brown-like phenotype whereas higher doses.

## Introduction

The increasing prevalence of obesity and its associated co-morbidities globally draws attention to the need for developing effective treatment or prevention strategies. Capsaicin, a bioactive component of chili peppers and a *TRPV1* agonist, has been reported to promote the process of thermogenesis [1], [2]. There is an established link between capsaicin ingestion and body weight regulation where studies indicate that capsaicin suppresses appetite and increases thermogenesis as well as energy expenditure in both rodents and human. Rats fed a diet containing 0.014% capsaicin showed significant reduction in visceral fat weight [1]. In long term feeding experiments, dietary capsaicin administration was reported to prevent weight gain in wild type mice but not in *TRPV1* knockout (KO) animals [3]. In humans, a meta-analysis of 20 trials found a modest benefit of capsaicin on weight loss via an increase in energy expenditure [4]. Further, capsaicin was also reported to boost thermogenesis in 50% healthy volunteers with no effect on satiety [5].

Rodents with chemical ablation (using high dose of capsaicin) of *TRPV1* containing sensory neurons had increased appetite [6] whereas when fed a high HFD, *TRPV1* KO animals accumulated less visceral fat [7]. Adding to the confusion, a recent study found no difference in weight gain between *TRPV1* KO and WT mice kept on HFD [8]. It is also speculated that the beneficial effects of *TRPV1* modulators, especially capsaicin, are preventive rather than therapeutic. Molecular approaches have demonstrated decreased *TRPV1* expression in visceral adipose tissue of both obese humans and mice as compared with their lean counterparts [3]. The non pungent analogue of capsaicin *i.e.* evodiamine also boosted energy consumption and prevented weight gain in HFD fed mice [9] and obese humans via multiple mechanisms [10]. Recently, it has been shown that capsaicin and its non-pungent analog, capsiate can activate BAT via either *TRPV1* activation or sympathetic/adrenergic stimulation [11], [12]. *TRPV1* neurons co-express with SP, CGRP and are modulated by NGF, NPY and BDNF [13], [14]. Given that these peptides have significant roles to play in weight gain and energy expenditure, one cannot rule out the potential effects of their interactions with *TRPV1*.

Approaches to augment “brite” cell population in WAT (*browning*) are gaining significant importance. It has been reported that various KO strains that resist body weight gain on HFD have higher number of “brite” cells [15], [16]. Advances have been made to understand and study pharmacological and nutritional/dietary agents as well as the signalling pathways that can contribute to *browning* of WAT. Some pharmacological agents that can promote *browning* are sympathetic activators like BDNF and leptin, prostaglandins like PGE2 and PGI2, cardiac natriuretic peptides and neuropeptides, *PPAR* (both *γ* and *α*) modulators, some hormones like irisin and FGF-2. These agents act through different mechanisms [17]. Numerous nutritional and dietary factors have been linked with *browning* including dietary methionine/leucine restriction, maternal under-nutrition and high fat/calorie diet-induced sympathetic inputs to adipose tissue, dietary chemicals such as fucoxanthin, olive oil constituents, conjugated linoleic acid, PUFA from marine sources, resveratrol, capsaicin and its analogue as well as others [17].

The exact mechanism of action of capsaicin is controversial i.e. whether *TRPV1* agonism, *TRPV1* or capsaicin sensitive neuron desensitization/blockade or browning of WAT or any other mechanism independent of *TRPV1*/direct action is responsible for its effect. A better understanding of the role of capsaicin, *TRPV1* and their interplay is warranted. Herein, we investigated in detail the anti-adipogenic effect of capsaicin and the modulatory role of *TRPV1* receptors in adipogenesis using *in vitro* and *in vivo* model systems.

## Results

### Capsaicin inhibits lipid accumulation in 3T3-L1 pre-adipocytes via *TRPV1* modulation

**Figure 1:**
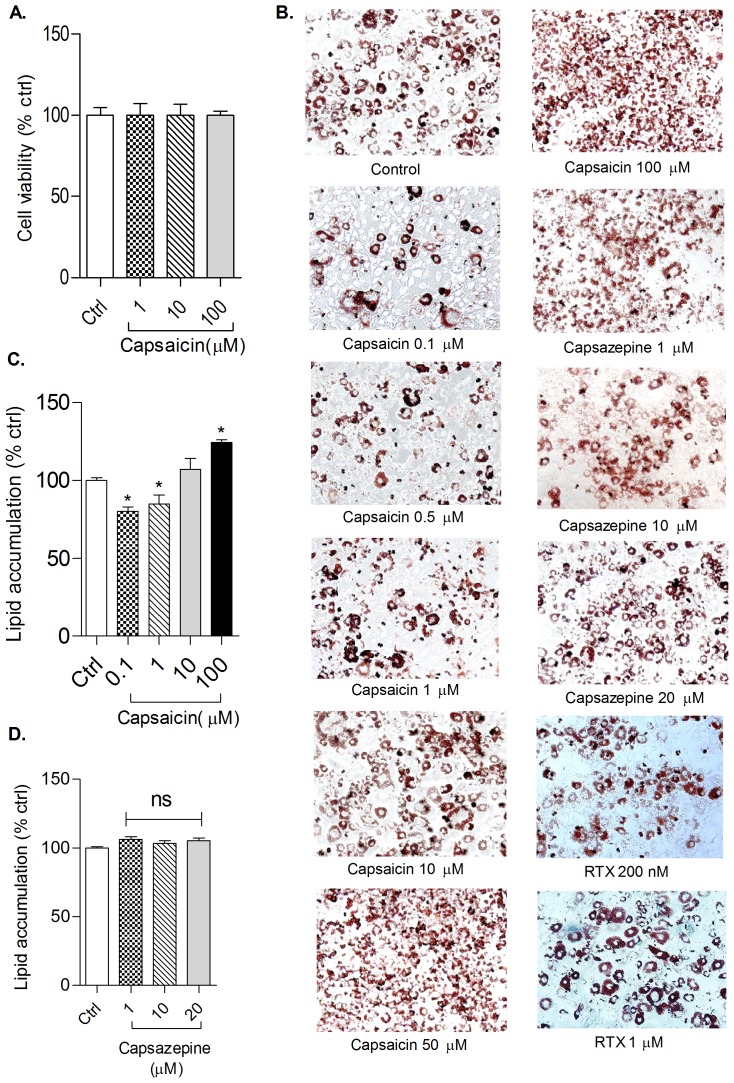
Effect of capsaicin and capsazepine on adipogenesis in 3T3-L1 cells. (A) Cell viability in pre-adipocytes treated with capsaicin for 72 hrs; (B) Effect of capsaicin, capsazepine and RTX on differentiation of 3T3-L1 cells. Black spots in images represents area stained by ORO dye; (C&D) Effect of capsaicin and capsazepine on lipid accumulation in 3T3-L1 adipocytes. All values are expressed as mean ± S.E.M. (n = 3). One way ANOVA followed by Tukey's multiple comparison post hoc test was applied. *P<0.05 as compared to control. ORO =  Oil red O, RTX =  resiniferatoxin.

In the present study, the anti-adipogenic effect of *TRPV1* agonist, capsaicin, was evaluated during adipogenesis. The cytotoxic effect of capsaicin (1, 10 and 100 µM) in pre-adipocytes was determined using MTT assay at different doses. There was no significant decrease in viability at the tested concentration (**Figure 1A**). Confluent 3T3-L1 pre-adipocytes were differentiated in presence of different doses of capsaicin (0.1, 0.5, 1, 10, 50 and 100 µM), RTX (200 nM and 1 µM) and capsazepine (1, 10 and 20 µM) in the differentiation media followed by maintenance media. Capsaicin at 0.1 and 1 µM significantly inhibited lipid accumulation whereas at 50 and 100 µM, lipid accumulation was significantly increased as compared to control (**Figure 1B and 1C**).

**Figure 2:**
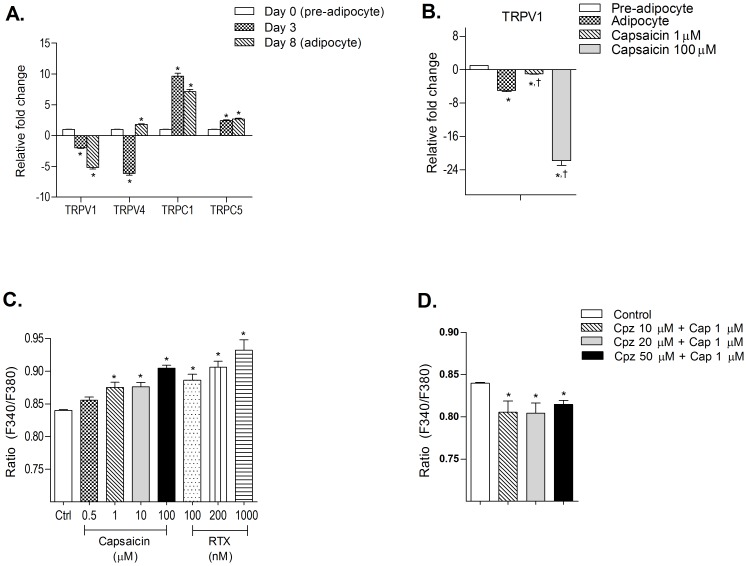
Effect of capsaicin on TRP expression and Ca^2+^ influx in 3T3-L1 cells. (A) Normalized mRNA expression levels of *TRPV1*, *TRPV4*, *TRPC1* and *TRPC5* in 3T3-L1 cells differentiated for 8 days; (B) Change in *TRPV1* expression levels in adipocyte when differentiated in presence of capsaicin. (C & D) Effect of *TRPV1* agonist, Cap and RTX, and antagonist, Cpz, on Ca^2+^ influx in pre-adipocytes. All values are expressed as mean ± S.E.M. (n = 3). *P<0.05 as compared to control or pre-adipocyte (wherever mentioned in panel), ^†^P<0.05 as compared to adipocyte. TRPV =  transient receptor potential channel vanaloid type, TRPC =  transient receptor potential channel canonical type, Cap =  Capsaicin, RTX =  resiniferatoxin, Cpz =  Capsazepine.

Expression levels of selected TRP channels were studied in pre-adipocytes and in adipocytes at early and late phases of adipocyte differentiation (**Figure 2A**). *TRPV1* was expressed in preadipocytes whereas its expression was down-regulated with the progression of adipogenesis with minimal expression at mature adipocyte stage.*TRPV4* showed moderate expression in preadipocytes. Expression was significantly down-regulated in cells at early adipogenesis whereas it was up-regulated at late adipogenesis phase. *TRPC1* showed moderate expression in preadipocytes and high expression in cells at early and late adipogenesis. *TRPC5* showed moderate expression in preadipocytes but expression was significantly increased with the progression of differentiation.

Capsaicin, at 1 µM significantly increased the expression levels of *TRPV1* in adipocytes whereas at 100 µM, the expression was significantly reduced compared with control adipocytes (**Figure 2B**). Further to confirm that inhibition of adipogenesis occurred *via* modulation of *TRPV1* channel, the effect of RTX, an ultra-potent *TRPV1* agonist, was studied on lipid accumulation during adipogenesis. An effect similar to that of capsaicin was observed *i.e.* at lower dose (200 nM) RTX inhibited lipid accumulation whereas at higher dose (1 µM) there was an increase in lipid accumulation (**Figure 1B**). No significant difference was observed in lipid accumulation at tested concentrations of *TRPV1* antagonist, capsazepine, as compared to control (**Figure 1B and 1D**).

Capsaicin and RTX showed dose-dependent increase in calcium influx in 3T3-L1 preadipocytes (**Figure 2C**). However, the effect of capsaicin (1 µM) was significantly reduced when cells were pre-treated with *TRPV1* antagonist capsazepine (**Figure 2D**).

### Effect of capsaicin on *PPARγ* and its downstream targets

**Figure 3:**
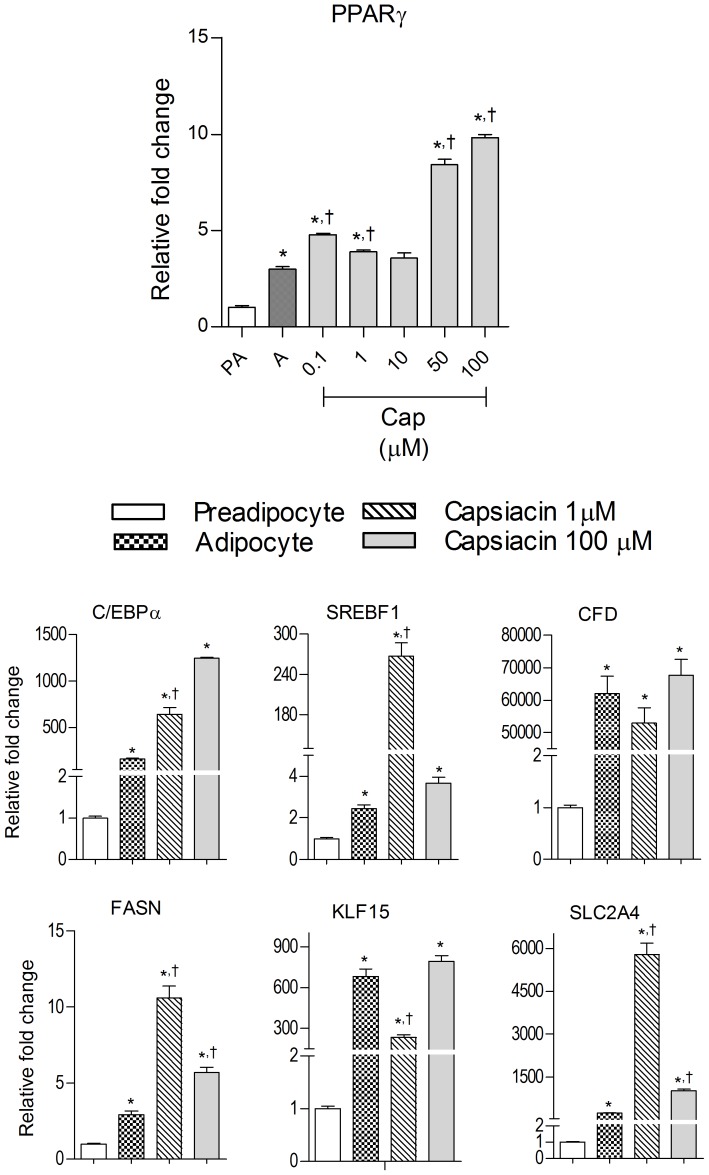
Effect of capsaicin on the expression of *PPARγ* and its downstream target genes. Data are expressed as mean ±S.E.M. (n = 3). *P<0.05 as compared to pre-adipocytes, ^†^P<0.05 as compared to adipocytes. PA =  Pre-adipocyte, A =  Adipocyte.

*PPARγ* was expressed more in adipocytes compared to preadipocytes. Its level was significantly enhanced at 0.1,1, 50 and 100 µM of capsaicin although the increase in expression at 50 and100 µM is many folds greater as compared to 0.1 and 1 µM. Similarly, the expression levels of *PPARγ* target genes such as *C/EBPα, SREBP1, CFD, FASN, KLF15* and *SLC2A4* were significantly high in adipocytes (**Figure 3**). The expression of *C/EBPα*, *SERBF1*, *FASN* & *SLC2A4* was significantly up-regulated by 1 µM capsaicin whereas no significant change was observed with 100 µM capsaicin except for *C/EBPα*, which showed marked increase in expression when compared to adipocytes. On the other hand, expression of *CFD* & *KLF15* was significantly decreased at 1 µM capsaicin, but no significant change was observed at 100 µM compared with control adipocytes (**Figure 3**).

### Capsaicin induces anti-adipogenic gene expression

**Figure 4:**
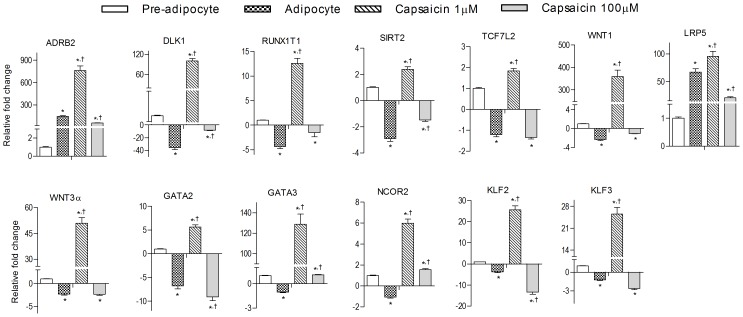
Effect of capsaicin on the expression of anti-adipogenesis genes during 3T3-L1 preadipocyte differentiation into adipocytes. Data are expressed as mean ± S.E.M., (n = 3). *P<0.05 as compared to pre-adipocytes, ^†^P<0.05 as compared to adipocytes.

Anti-adipogenic genes were evaluated in pre-adipocyte, untreated adipocytes and in adipocytes formed in the presence of 1 &100 µM of capsaicin in order to understand the underlying mechanism of adipogenesis inhibition. In mature adipocytes, genes such as *DLK1, NCOR2, RUNX1T1, TCF7L2*, *WNT1, WNT3A, GATA-2, GATA-3, KLF2* and *KLF3* were significantly down-regulated whereas *ADRB2* and *LRP5* were moderately expressed as compared to preadipocytes. Adipocytes treated with capsaicin showed significant increased expression of these genes at lower dose (1 µM) and significantly lowered expression at higher capsaicin dose (100 µM) (**Figure 4**).

### Capsaicin induces brown adipocyte specific markers in 3T3-L1 cells

**Figure 5:**
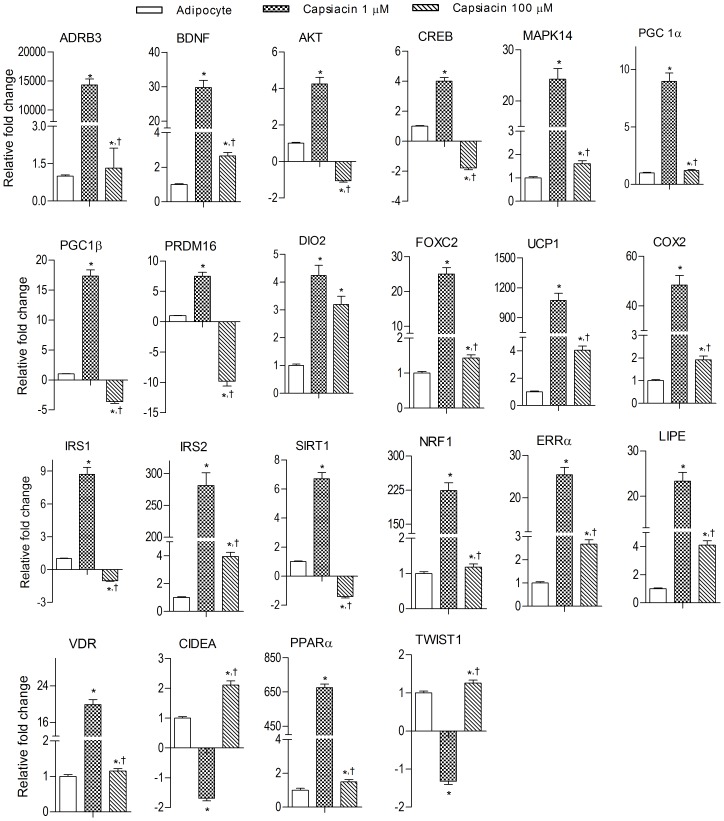
Effect of capsaicin on brown adipocyte specific genes in 3T3-L1 adipocytes. Data are shown mean ± S.E.M., (n = 3). *P<0.05 as compared to adipocytes, ^†^P<0.05 as compared to capsaicin (1 µM).

*PPARγ* activation promotes brown adipocyte-like phenotype in white adipocytes via induction of brown-specific genes, such as *UCP*1, *PGC-1α* [18]. We observed increased expression of *PPARγ* in the presence of capsaicin, hence we analyzed the expression of various transcription factors associated with browning of white adipocytes including, among others, *UCP1, PGC1α, PRDM16, DIO2, PPARα*, and *FOXC2* (**Figure 5**). The expression levels of these genes were significantly decreased during the differentiation of 3T3-L1 preadipocyte. Low dose (1 µM) capsaicin treatment enhanced expression significantly while higher dose (100 µM) capsaicin treatment had the opposite effect. Also genes like *CIDEA* and *TWIST1* that are known to negatively regulate the expression of *UCP1* and *PGC1α*, respectively, were inhibited at 1 µM whereas the effect was reversed at 100 µM. However, increased expression of *VDR*, the negative regulator of *UCP1*, was observed at capsaicin 1 µM.

### Effect of co-administration of capsaicin and capsazepine on *PPARγ*, adipogenesis modulating genes and brown adipocyte specific markers

**Figure 6:**
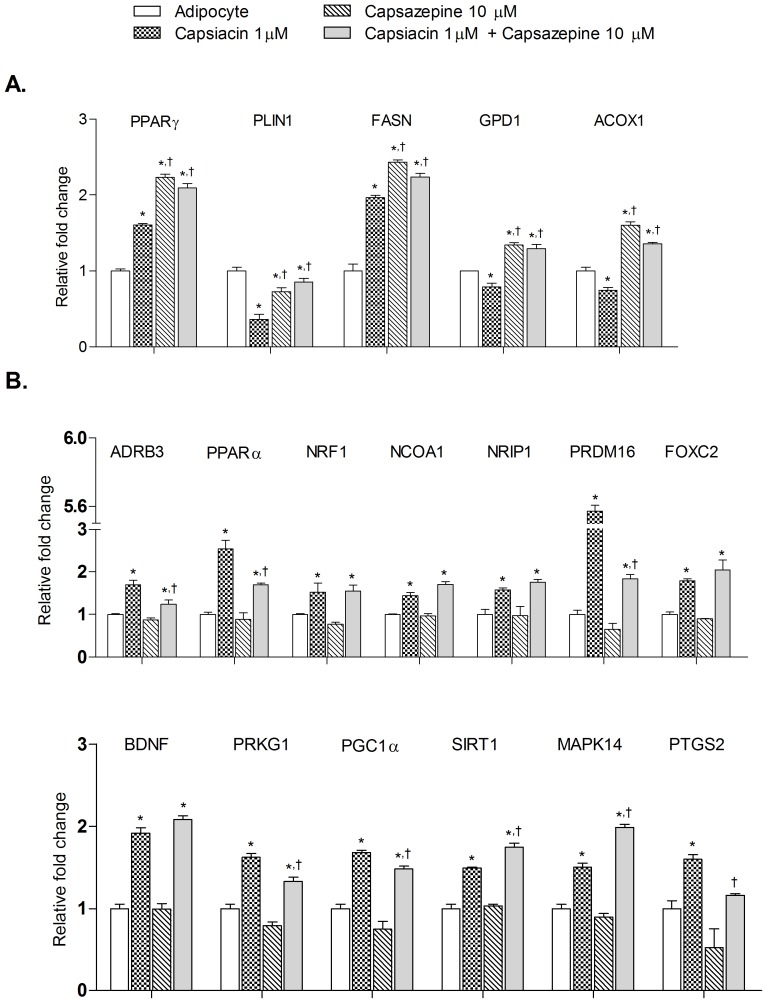
Effect of co-administration of capsaicin and capsazepine on *PPAR γ*, adipogenesis modulating genes and brown adipocyte specific genes in 3T3-L1 adipocytes. All values are presented as mean ± S.E.M. (n = 3). *P<0.05 as compared to adipocyte, ^†^P<0.05 as compared to capsaicin (1 µM).

Capsaicin significantly enhanced the expression of *PPARγ* and *FASN* in adipocytes and expression levels were further enhanced with capsazepine treatment (**Figure 6A**). Metabolic genes such as *PLIN1, GPD1* and *ACOX1* showed the opposite trend with their expression level being significantly lowered by capsaicin treatment whereas significantly enhanced in capsazepine treated group. Co-administration of capsaicin and capsazepine showed capsazepine like changes in expression pattern, suggesting inhibition of *TRPV1* activity (**Figure 6A**).

We also analyzed the expression of various thermogenic genes and genes associated with “*browning*” in white adipocytes including, among others, *BDNF, PGC1α, NCOA1, FOXC2, PRDM16* and *SIRT1* (**Figure 6B**). Their expression levels were significantly increased by capsaicin (1 µM) whereas capsazepine (10 µM) did not show any significant effect. On the other hand, co-administration of capsaicin (1 µM) and capsazepine (10 µM) showed differential effect. Expression levels of genes such as *ADRB3*, *PPARα, PRDM16, PRKG1, PGC1α* and *PTGS2* were significantly decreased whereas genes such as *SIRT1* and *MAPK14* showed significantly higher expression as compared to capsaicin treatment group. Also genes such as NRF1, *NCOA1* NRIP1, FOXC2 and BDNF showed no significant change in expression as compared to capsaicin treatment group.

### Effect of capsaicin on adipokine secretion

**Figure 7:**
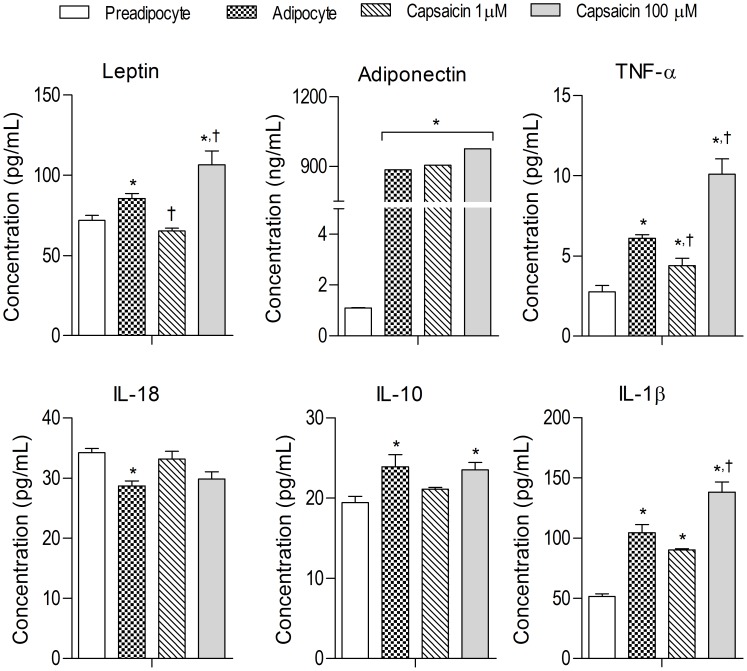
Effect of capsaicin on the release of inflammatory mediators during 3T3-L1 preadipocyte differentiation into adipocytes. All values are presented as mean ± S.E.M. (n = 3). *P<0.05 as compared to pre-adipocytes, ^†^P<0.05 as compared to adipocytes.

Leptin secretion was enhanced in adipocytes treated with 100 µM capsaicin. However, secretion was significantly inhibited at 1 µM concentration of capsaicin. Interestingly, adipokine secretion was stimulates by high dose of capsaicin (100 µM) while low dose of capsaicin (1 µM) showed no effect. Release of pro-inflammatory molecules such as *TNFα* and *IL-1β*, was lowered by capsaicin (1 µM) whereas the opposite effect was observed at 100 µM dose. Simultaneously, increased levels of *IL-18*, a pro-inflammatory mediator, was observed at 1 µM capsaicin. *IL-10*, an anti-inflammatory molecule was inhibited at the lower dose of capsaicin while elevated levels were observed at the higher dose (**Figure 7**).

### Effect of *TRPV1* ablation on body weight, thermal pain sensitivity and locomotor activity in normal and HFD fed animals

**Figure 8:**
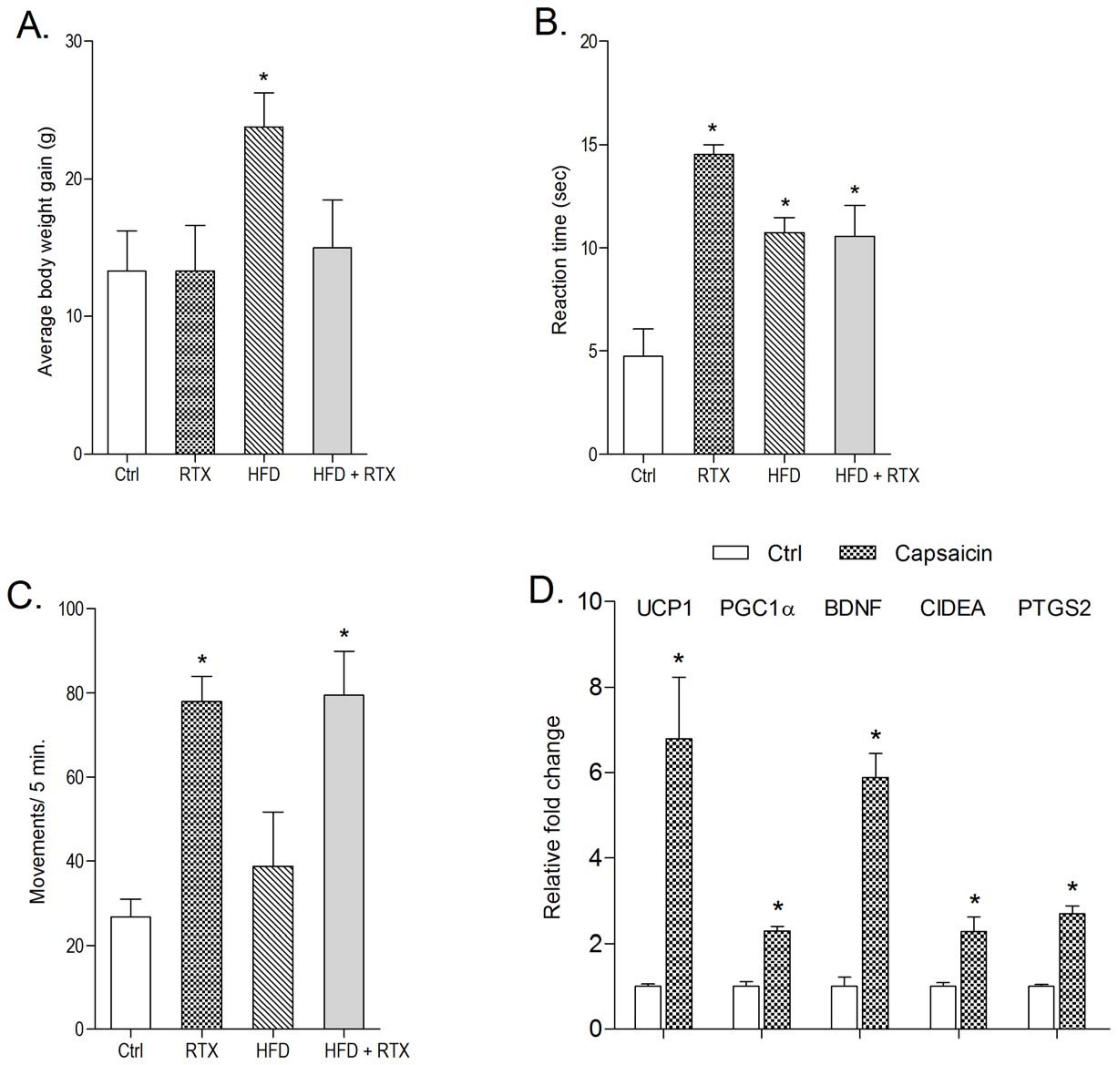
Effect of *TRPV1* ablation on: (A) Average body weight gain after 4 weeks, (B) pain threshold and (C) locomoter activity (in photoactometer) after one week. (D) Effect of capsaicin on brown adipocyte specific genes in sWAT. All values are expressed as mean ± S.E.M., (n = 5). *P<0.05 as compared to control.

One week after RTX administration, reaction time at hot plate test was significantly increased, suggesting an ablation of *TRPV1* (**Figure 8B**). RTX administered rats resisted weight gain on a HFD (45% fat) (**Figure 8A**). Furthermore, in both chow and HFD fed animals, locomotor activity was seen to be increased significantly upon RTX administration (**Figure 8C**). No change in anxiety response was observed (data not shown).

### Effect of capsaicin supplementation on brown adipocyte specific markers in subcutaneous WAT (sWAT)

Capsaicin's effect on selected thermogenic and related genes such as *UCP1*, *PGC1α*, *BDNF*, *CIDEA* and *PTGS2* in sWAT was evaluated. The expression of these genes was significantly up-regulated upon capsaicin administration (2 mg/kg, p.o. for 3 months) as compared to control animals (**Figure 8D**).

## Discussion

The present study gives an insight into the modulatory role of *TRPV1* and the dose dependent effect of capsaicin on the differentiation of 3T3-L1 preadipocyte cells into adipocytes. We provide evidence showing the dual effect of capsaicin *i.e.* inhibition of adipogenesis at lower dose (0.1–1 µM) and stimulation of adipogenesis at higher dose (10–100 µM). The anti-adipogenic effect of capsaicin was accompanied with induction of brown-like phenotype in 3T3-L1 adipocytes.

Reports on *TRPV1* expression in adipocytes are contradictory. Zhang *et al.* had reported significant expression of *TRPV1* in 3T3-L1 preadipocytes and adipocytes (less than preadipocytes) from obese (*ob/ob* and *db/db*) mice and humans suggesting the functional role of *TRPV1* in obesity and adipogenesis [3]. On the contrary, electrophysiology and calcium imaging studies failed to detect the functional expression of *TRPV1* in 3T3-L1 preadipocytes [7] and mouse adipocytes [19]. In the present study, moderate expression of *TRPV1* in 3T3-L1 preadipocytes was detected which was significantly decreased (upto minimum/non functional levels, as suggested by calcium imaging) with their differentiation into adipocytes. At the same time *TRPV4*, *TRPC1* and *TRPC5* showed higher expression in preadipocytes as well as in differentiated adipocytes, which is in accordance with existing literature [19], [20]. Capsaicin at low dose (0.1–1 µM) inhibited lipid accumulation in adipocytes while at higher dose (10–100 µM) it enhanced lipid accumulation. Expression levels of *TRPV1* were significantly enhanced at lower dose of capsaicin (1 µM) whereas at 100 µM concentration, its expression was significantly decreased. Given the absence of *TRPV1* expression in adipocytes, we can argue here, that *TRPV1* expression detected is from preadipocytes. Since, capsaicin at lower concentration (1 µM) inhibited lipid accumulation during differentiation; hence we may speculate that there is an increased % of preadipocytes. To determine whether the effects are *TRPV1* dependent or not, the effects of different doses of RTX, a *TRPV1* agonist (200 nM and 1 µM) and capsazepine, a *TRPV1* antagonist (1–20 µM) were studied on lipid accumulation. In RTX treated group, we observed a pattern similar to that of capsaicin, less lipid accumulation on lower dose and increased lipid accumulation at higher dose. Capsazepine *per se* did not significantly alter lipid accumulation during differentiation. Further, both capsaicin and RTX (*TRPV1* agonists) dose dependently increased Ca^2+^ influx in preadipocytes suggesting functional presence of *TRPV1*. However, in the presence of capsazepine, the capsaicin-induced increase in Ca^2+^ influx in preadipocytes was significantly reduced. In case of adipocytes we failed to observe any Ca^2+^ influx following the administration of these agents (data not shown). Hence, we may conclude that lower doses of *TRPV1* agonists inhibit adipogenesis whereas higher doses stimulate adipogenesis. We believe that at higher concentration both capsaicin and RTX may decrease receptor activity due to desensitization/internalization of receptor or by killing the cell due to abrupt calcium influx [21]. However, that does not seem to be the case since in the presence of multiple doses of capsazepine (*TRPV1* inhibition, receptor inactivity), there was only a slight statistically non-significant increase in adipogenesis. Hence, our studies suggest that *TRPV1* plays a modulatory role in preadipocyte differentiation but has a limited role in the increase in adipogenesis seen at higher doses of *TRPV1* agonists. It is possible that there may be non-*TRPV1* dependent mechanisms to explain the same.

Further, we looked into the gene expression profile of 3T3-L1 pre-adipocytes and adipocytes formed in the presence of capsaicin. Earlier reports suggested that capsaicin affects adipogenesis regulating genes (*PPARγ*, *C/EBPα*, *aP2*) and genes involved in lipid metabolism (*HSL, CPTI-α, UCP2, GPDH* and leptin) [22], [23]. There are multiple reports suggesting the direct action of capsaicin on *PPARγ* activity [24], [25]. Increasing concentration of capsaicin (0.1–100 µM) led to increased expression of *PPARγ*, with 100 µM capsaicin showing significantly elevated expression of *PPARγ*. It is quite possible that the adipogenic activity of capsaicin at higher doses may be due to high levels of *PPARγ* [24], [26]. Although there are no conclusive proofs but we may argue that increase in *PPARγ* activity beyond a threshold may promote adipogenesis, hence there is differential effect of capsaicin at different doses. We tried to understand the role of *PPARγ* by studing the expression pattern of *PPARγ* targets like *C/EBPα, SREBF1, FASN, SLC2A4, CFD* and *KLF15*. We were unable to find any correlation between the expression of *PPARγ* and its target genes. Involvement of these genes in multiple physiological functions and their dependence on various endogenous and exogenous factors may contribute to the observed lack of correlation. But this forced us to think that non-*TRPV1* dependent effects of capsaicin might be playing a significant role, especially at higher doses of capsaicin. Capsazepine (10 µM) showed an effect opposite to capsaicin and also prevented capsaicin-induced change in gene expression pattern of *PPARγ* and its target genes on co-administration.

Looking at the rather in-conclusive pattern of expression of pro-adipogenic genes, we looked at anti-adipogenic genes as well, which have shown quite consistent results. Thirteen anti-adipogenic genes were identified and most showed decreased expression during adipogenesis while only two genes, *ADRB2* and *LRP5*, showed higher expression. These genes function via multiple pathways whose enhanced expression upon capsaicin treatment resulted in inhibition of adipogenesis. Some genes like *NCOR2, SIRT2, GATA2, GATA3, KLF2* act via targeting *PPARγ*, a major regulator of adipogenesis [27], [28], [29], [30]. Others like *TCF7L2, WNT1, WNT3A* and *LRP5* have a role in Wnt/β-catenin pathway [31], [32] whereas *DLK1* activates MAPK kinase/ERK pathway [33] and *ADRB2* regulate adipogenesis via cAMP/PKA pathway [34]. Adipogenesis is a complex process and numerous genes and pathways are involved. It is possible that a few of them show opposite trends due to the association of these genes with other cell survival functions along with differentiation. Capsaicin (1 µM) significantly enhanced all these genes as compared to control adipocytes. Genes such as *TCF7L2, WNT3A, GATA2, KLF2*, *KLF3* whose expression, relative to control adipocytes, was significantly reduced by the effect of capsaicin (100 µM) might play a more prominent role in adipogenesis whereas few others like *DLK1, RUNX1T1, SIRT2, WNT1, GATA3, LRP5, and ADRB2* seem less involved.

Due to changes in the gene expression of *PPARγ*, we hypothesized that capsaicin may play a role in developing “brite” cells or brown phenotype within white adipocytes. Therefore, we investigated the effect of capsaicin on “*browning*” specific genes by targeting brown-specific genes responsible for formation of proteins that show their activity right from the cell membrane to the nucleus. Induction of brown like phenotype upon capsaicin treatment is associated with a number of genes and pathways. Adrenergic stimulation plays an important role in activation of *UCP1*, the hallmark protein for brown adipocytes. It involves a number of genes such as *β-ARs, p38 MAPK, DIO2, PGC-1α*, that were up-regulated by capsaicin (1 µM). mRNA levels of BDNF, the neuropeptide that induces brown fat marker genes in WAT, were increased in capsaicin treated cells. Many other genes like PPAR*δ*, *C/EBPβ, SIRT1, MAPK14, CREB, PRDM16* can directly activate *PGC1α* gene expression which is the principal regulator of genes involved in mitochondrial biogenesis, lipid metabolism and thermogenesis, and mediates its effect by targeting its downstream genes such as *NRF1, NRF2, ERRα, PPARα, PPARγ, UCP1*. Genes negatively regulating expression of *PGC1α* and *UCP1* (*i.e. TWIST, CIDEA*) were lowered by capsaicin (1 µM). Increase in cytoplasmic Ca^2+^ levels is also responsible for *PGC1α* activation. It may be possible that activation of *TRPV1* has direct effect on *PGC1α* activation due to Ca^2+^ influx. Capsazepine (10 µM) showed an effect opposite to capsaicin and also prevented capsaicin-induced changes in gene expression pattern of important “browning” (*PRDM16, CIDEA, PGC-1α*) genes. Interestingly, at higher doses, capsaicin did not induce “browning” phenotype. The substantially higher values of *PPARγ* at higher doses of capsaicin may be responsible; we believe that once the threshold is crossed adipogenesis is stimulated which will limit the “*browning*” effect. The interrelationship between capsaicin doses, *PPARγ* expression and induction of “*browning*” may need a deeper understanding and further experimental evidence for its physiological relevance. Further, in LACA we observed a significant increase in the transcriptional levels of “brite” specific genes in WAT.

Further, we have studied the influence of capsasicin administration on the functional release of pro/anti inflammatory mediators and hormones. Levels of leptin, adiponectin, anti-inflammatory molecule (*IL-10*) and pro-inflammatory mediators (*TNF-α* and *IL-1β* but not *IL-18*) were up-regulated in adipocytes. Leptin and adiponectin are the two major cytokines secreted by adipocytes. Leptin regulates energy expenditure and energy intake *via* its action in the brain whereas adiponectin has a role in lipid metabolism in adipocytes [35], [36]. Various studies suggest that pro-inflammatory cytokines (*TNFα, IL-18* and *IL-1β*) suppress adiponectin expression [37], [38], [39] and influence leptin levels [40]. *IL-10* is a potent anti-inflammatory cytokine secreted by adipocytes to inhibit inflammatory responses [41]. In the present study, capsaicin at 1 µM significantly decreased the levels of pro-inflammatory markers *i.e. TNFα* and *IL-1β*, whereas *IL-18* levels were increased. As adiponectin levels remained unchanged; this could be attributed to the combined actions of these inflammatory mediators. Levels of *IL-10* were also inhibited by capsaicin at 1 µM which may be due to the inhibition of adipogenesis at this concentration. The possible role of *TRPV1* activation and Ca^2+^ influx on the vesicular release of these mediators cannot be ruled out. Hence, interplay between adipogenesis, receptor activity and inflammation plays a role in the overall capsaicin-induced effects. In our study, the release of adiponectin is not affected by capsaicin administration which may be due to dual role of adiponectin in adipogenesis. It has both pro-inflammatory [42], [43] and anti-inflammatory roles [41], [44].

Based on *in-vitro* results, we speculate that *TRPV1* activation in *in-vivo* system will also lead to inhibition of adipogenesis which is very well proven using multiple animal models [3],[45]. Studies with *TRPV1* KO animals suggest that knock down of *TRPV1* actually prevents mice from becoming obese [7]. We used intraperitoneal administration of RTX in order to selectively ablate *TRPV1* containing neurons throughout, hence mimicking *TRPV1* knockout animals [46], [13]. *TRPV1* ablation was confirmed by thermal pain sensitivity testing *via* hot plate where RTX treated animals showed a significant decrease in paw withdrawal latency. We have demonstrated the effect of *TRPV1* ablation by RTX on body weight gain and locomotor activity. We observed (a) no significant increase in body weight on a HFD and (b) an increase in locomotor activity after *TRPV1* ablation. Increase in locomotor activity may be related with energy expenditure and hence limited weight gain and even cause some reduction. Ablation of *TRPV1* is also associated with hyperthermia, which is related to heat/energy loss and reduction in weight [47]. Here, we may argue that at higher doses of capsaicin where *TRPV1* activity is non-functional, the effect is due to *TRPV1* independent mechanisms.

In summary, the study provides evidence that capsaicin has a dual modulatory role in adipogenesis. Capsaicin inhibited adipogenesis and induced brown-like phenotype at lower doses *via TRPV1* dependent mechanism whereas adipogenesis was promoted at higher dose by *TRPV1* independent mechanisms.

## Materials and Methods

### Reagents

Mouse 3T3-L1 preadipocyte cells were obtained from Zenbio Inc. (Research Triangle Park, NC). Dulbecco's modified Eagle's medium (DMEM), 3T3-L1 adipocyte differentiation media (DM-2-L1) and 3T3-L1 adipocyte maintenance media (AM-1-L1) were obtained from Zenbio Inc. (Research Triangle Park, NC). Fetal calf serum (FCS), penicillin-streptomycin, trypsin-EDTA solution, trypan blue solution (0.4%), Oil Red O (ORO) dye, MTT assay kit, capsaicin (≥95%) and resiniferatoxin were purchased from Sigma-Aldrich, Inc. (St. Louis, MO, USA). Regular low-fat diet (D12450B) and high-fat diet (D12492) were purchased from Research Diets (New Brunswick, NJ, USA). All other reagents used were of analytical grade and obtained from local suppliers. Stock solution of capsaicin and RTX were prepared in absolute ethanol.

### 3T3-L1 Cell culture

3T3-L1 preadipocytes were propagated in high glucose DMEM supplemented with 10% (v/v) FCS and 1% penicillin-streptomycin at 37°C for 48 h in a humidified atmosphere having 5% CO_2_. Differentiation was induced in confluent cells by replacing DMEM with differentiation media. After 2 days, cells were switched to the maintenance media for another 8 days with media replacement on every second day. For capsaicin, capsazepine and RTX treatment, two day confluent pre-adipocytes were incubated with different doses of capsaicin (0.1, 0.5, 1, 10, 50 and 100 µM), capsazepine (1, 10 and 20 µM) and RTX (200 nM and 1 µM) during the differentiation and till mature adipocyte formation. Cells treated with ethanol (1∶1000 dilution) during differentiation and maintenance were used as a control.

### Cell viability assay

Pre-adipocytes were seeded in a 96-well plate at a density of 1×10^4^ cells/well and incubated until confluence. Cells were treated with different concentrations of capsaicin (1, 10 and 100 µM) for 72 h. Preadipocytes treated with ethanol (1∶1000) were used as control. After 72 h, media was removed and treated with MTT as per manufacturer's instruction. Absorbance was measured at 570 nm, with background subtraction at 690 nm, using a microplate reader (Spectra Max M5^e^, Molecular Devices, Minnesota, USA). Six replicate wells were used for each data point in the experiment.

### Oil red O staining

Pre-adipocytes were seeded in a 96-well plate at a density of 1×10^4^ cells per well and allowed to reach confluence. The cells were treated with different doses of capsaicin (0.1, 0.5, 1, 10, 50 and 100 µM), RTX (200 nM and 1 µM) and capsazepine (1, 10 and 20 µM) in the differentiation and maintenance medium. Ethanol (1∶1000) treated cells were used as a control. Intracellular lipid accumulation was quantified using ORO staining [48]. The stained lipid droplets were visualized using an inverted microscope. Intracellular lipid content was quantified after extracting ORO bound to cells with 100% isopropanol and absorbance at 500 nm was determined in six replicate wells using a microplate reader.

### Intracellular calcium measurements

Preadipocytes were grown in DMEM with 10% (v/v) FCS and 1% penicillin-streptomycin. To measure the intracellular Ca^2+^ levels in undifferentiated 3T3-L1 cells, the media was replaced with serum-free DMEM. Fluorophore, fura-2 AM (Molecular Probes Inc, Eugene, OR), was added at a final concentration of 10 µM to each well and the cells were incubated for 45 minutes in dark at 37 °C (5% CO_2_). Fluorescence due to intracellular calcium was measured at 510 nm emission, 340 nm and 380 nm excitation wavelengths using a spectraMax M5e microplate reader. Intracellular calcium concentrations were calculated from the ratio of transient increase in fluorescence intensity at 340 nm and 380 nm. After establishment of a stable baseline, the response to *TRPV1* agonists, *i.e.* capsaicin (0.5, 1, 10 and 100 µM) and RTX (100, 200 and 1000 nM), and combination of capsaicin (1 µM) with different does of *TRPV1* antagonist, capsazepine (1, 10 and 20 µM), was determined.

### Adipokine secretion by 3T3-L1 adipocytes

Supernatants collected, on the final day of treatment, were used to quantify adipokines such as leptin, adiponectin, interleukin-10 (*IL-10*), interleukin-1 beta (*IL-1β*), interleukin-18 (*IL-18*) and tumour necrosis factor-alpha (*TNF-α*) by ELISA using commercially available kits as per manufacturer's instructions. Supernatant from preadipocytes was also taken as a control. All commercial mouse ELISA kits used above were purchased from Invitrogen (Camarillo, CA).

### Total RNA extraction

Preadipocytes were cultured in 6-well cell culture plates and incubated until confluence. Differentiation was initiated by addition of differentiation media and after 48 h, it was replaced with maintenance media (changed every 2 days). Cells were harvested at different time points during adipogenesis *i.e.* at preadipocyte stage (day 2), two days after induction of differentiation (day 4) and after complete maturation of adipocytes (day10). Simultaneously, cells were also treated with 0.1, 1, 10, 50 and 100 µM of capsaicin, 10 µM of capsazepine and combination of capsaicin (1 µM) and capsazepine (10 µM) during the adipogenesis period (Days 0–10). On day 10, after maturation, cells were harvested. All the harvested cells were then used to extract total RNA using ribopure RNA extraction kit (Invitrogen, USA) as per the manufacturer's instructions. The quantitative and qualitative ratio metric analysis of RNA was done using Infinite M200 ProNanoQuant (Tecan, Switzerland). RNA integrity was confirmed using 1.4% agarose gel.

### cDNA synthesis and quantitative PCR

cDNA was synthesized from RNA (1 µg) using single strand cDNA synthesis kit (Qiagen, USA) as per manufacturer's instructions. Relative expression levels of different transient receptor potential (TRP) channel genes during adipogenesis, expression levels of *TRPV1* gene (in capsaicin treated cells) and murine genes such as the anti-adipogenic genes (*i.e. ADRB2, NCOR2, TCF7L2, KLF2* etc.), *PPARγ* & their target genes (*i.e. C/EBP α, FASN* etc.) as well as brown adipocyte markers (*i.e.UCP1, PGC1α, PRDM16*) were determined by quantitative PCR (qPCR) (Applied Biosystems 7500 Fast Real-Time PCR machine) using SYBR green based custom designed PCR array: CAPM11591, PAMM-049ZA and CAPM11784 respectively (Custom Mouse RT^2^ profile PCR array, SABiosciences, Qiagen, USA). The conditions for RT-PCR were: 95°C for 10 minutes, followed by 40 cycles of 95°C and 60°C for 1 minute. Data was analyzed using ΔΔC_t_ method provided by SABiosciences, Qiagen, USA with normalization of adipogenic gene expression by the geometric mean of five housekeeping (*GAPDH, ACTB,HSP90AB1, HPRT, GUSB*) genes, brown marker genes expression by *18sRNA* and *PPARγ* & *TRPV1* gene expression by *GAPDH*.

### *In-vivo* experiments

Male Wistar rats (180–250 g), 3 months, bred in Central Animal House facility (CAH) of Panjab University, Chandigarh, India were used. Animals were housed under standard laboratory condition with 12 hours light dark cycle, with free access to regular low-fat diet and water. All the experimental protocols were approved by the Institutional Animal Ethical Committee (IAEC), Panjab University and conducted according to the Committee for the Purpose of Control and Supervision on Experiments on Animals (CPCSEA) guidelines on the use and care of experimental animals. After one week of acclimatization, rats were randomized (as per weight) and divided into the following four groups with (n = 5) in each group.

Group1: Control (normal diet containing 10% fat)

Group2: Control + RTX (200 µg/kg/ml)

Group3: HFD (45% fat)

Group4: HFD + RTX (200 µg/kg/ml)

Each rat in RTX group and RTX+HFD group received 200 µg/kg/ml of RTX (dissolved in normal saline with 3% ethanol and 10% tween 80). Control and HFD group received the corresponding vehicle. Doses were selected on the basis of previous literature available [49]. Body weights were measured every week starting from day 0. All the behavioural experiments (pain threshold [50], locomotor activity [51] and elevated plus maze [52]) were performed between 9:00 am and 4:00 pm.

In another set of experiments, male LACA mice (5–6 week old; 25±3 g) were used in the study. After one week acclimatization, animals were randomly divided into two groups: Normal diet (Ctrl) group and normal diet with capsaicin intervention (Cap) group. Each mice in Cap group orally received 2 mg/kg BW of capsaicin (dissolved in 0.9% saline with 3% ethanol and 10% tween 80) on alternate days for 12 weeks. Ctrl group received the corresponding vehicle. At the end of the feeding period animals were killed by cervical dislocation and sWAT was isolated and samples were snap frozen and stored at −80°C for subsequent RNA isolation and gene (*UCP-1, PGC1α, BDNF, CIDEA* and *PTGS2*) expression analysis.

### Statistical analysis

The data are expressed as mean ± S.E.M. Statistical analysis was performed using Prism Graphpad software (GraphPad Software Inc., CA, USA). Unless otherwise stated, one way analysis of variance (ANOVA) followed by Tukey's multiple comparison test was applied to check the level of significance. In all the tests, p<0.05 was taken as criterion for statistical significance.
